# 利用*β*-*N*-乙酰氨基葡萄糖苷酶快速酶切分析中国仓鼠卵巢细胞表达的西妥昔单抗抗原结合区的聚糖比例

**DOI:** 10.3724/SP.J.1123.2021.05008

**Published:** 2022-02-08

**Authors:** Qian CHENG, Daihui JIA, Bohui ZHANG, Junyan XU, Zhe SHAO, Yingfeng HUANG, Xun ZOU

**Affiliations:** 宝船生物医药科技(上海)有限公司, 上海 201203; Dragonboat Biopharmaceutical (Shanghai), Co., Ltd, Shanghai 201203, China; 宝船生物医药科技(上海)有限公司, 上海 201203; Dragonboat Biopharmaceutical (Shanghai), Co., Ltd, Shanghai 201203, China; 宝船生物医药科技(上海)有限公司, 上海 201203; Dragonboat Biopharmaceutical (Shanghai), Co., Ltd, Shanghai 201203, China; 宝船生物医药科技(上海)有限公司, 上海 201203; Dragonboat Biopharmaceutical (Shanghai), Co., Ltd, Shanghai 201203, China; 宝船生物医药科技(上海)有限公司, 上海 201203; Dragonboat Biopharmaceutical (Shanghai), Co., Ltd, Shanghai 201203, China; 宝船生物医药科技(上海)有限公司, 上海 201203; Dragonboat Biopharmaceutical (Shanghai), Co., Ltd, Shanghai 201203, China; 宝船生物医药科技(上海)有限公司, 上海 201203; Dragonboat Biopharmaceutical (Shanghai), Co., Ltd, Shanghai 201203, China

**Keywords:** 超高效液相色谱, 高分辨质谱, 糖基化, 糖苷酶, 西妥昔单抗, ultra performance liquid chromatography (UPLC), high resolution mass spectrometry (HRMS), glycosylation, glycosidase, cetuximab

## Abstract

西妥昔单抗具有较复杂的糖基化修饰,在抗原结合片段(Fab)和可结晶片段(Fc)的重链上都含有2个*N*-糖基化位点,其中Fab段的糖基化最为复杂,要研究清楚该位点的糖基化修饰,开发专一性切糖技术和稳定的聚糖比例分析方法是当前迫切需要解决的难题。以中国仓鼠卵巢(CHO)细胞表达的西妥昔单抗为研究对象,使用*β*-*N*-乙酰氨基葡萄糖苷酶(Endo F2)开发了一种快速Fab段聚糖释放的方法,利用超高效液相色谱-高分辨质谱(UPLC-HRMS)进行了定性和聚糖比例分析。第一步对抗体原液进行非变性酶切,抗体原液经超纯水稀释后,加入糖苷酶Endo F2进行酶切,通过质谱对质量数的解析,结果表明Endo F2酶切时间5 min, Fab段的聚糖就能完全切除,而Fc段的聚糖不受影响,实现了快速酶切,而且切糖具有很好的专一性。第二步对Fab段聚糖进行比例分析,将释放的聚糖经对氨基苯甲酰胺(2-AB)荧光标记后使用超高效液相色谱联用荧光检测器(FLR)进行检测,在亲水作用色谱(HILIC)柱上得到良好的分离并可以进行稳定地聚糖比例分析。3次独立试验结果表明,酶切后的质谱图基本一致,且聚糖的比例结果也基本一致,表明Endo F2酶切方法和聚糖比例分析方法都具有较好的稳定性和可靠性。此外,通过测定来自两个不同工艺生产的样品,数据显示两者的糖谱上具有非常明显的差异,表明利用开发的方法可以实现对抗体生产工艺进行监测研究,对抗体生产工艺的评估具有非常重要的意义。

## Abstract (en)

The *N*-glycosylation of proteins is a typical post-translational modification. Compared with other monoclonal antibodies, *N*-glycosylation modification in cetuximab is more complicated. Because cetuximab contains two *N*-glycosylation sites, one is located on the antigen-binding fragment (Fab) and the other is on the crystallizable fragment (Fc) of the heavy chain (HC). Among the two, the glycosylation of the Fab segment is more complicated. As this segment is located in the hypervariable region (VH), it may affect the affinity of the antibody antigen and cause other issues. Therefore, it is necessary to study glycosylation modification at this site. This modification is particularly challenging, necessitating the development of specific glycan cutting technology and a stable glycan ratio analysis method.

In this study, cetuximab expressed in Chinese hamster ovary (CHO) cell was used as the experimental research object. Based on the digestion with endo-*β*-*N*-acetylglucosaminidase F2 (Endo F2), an experimental method was developed that can quickly release Fab glycans. Qualitative and glycan ratio analyses were carried out by ultra performance liquid chromatography-high resolution mass spectrometry (UPLC-HRMS). The test was divided into two steps: in the first step, a non-denaturing (native state) glycosidase excision test was performed on the CHO-cetuximab drug substance. The drug substance was diluted to 1.0 mg/mL by adding ultrapure water, following which 1.0 μL of Endo F2 was directly added to 100 μL of the drug substance for enzyme digestion at 37 ℃. Through HRMS, the data were deconvoluted to obtain the accurate mass of the drug substance. The results showed that when the digestion time of Endo F2 was 5 min, the glycans in the Fab segment could be completely removed, whereas those in the Fc segment were not affected. Rapid enzyme cutting of the Fab glycans was realized; simultaneously, it was concluded that this method was also very specific for the removal of Fab glycans. In the second step, an accurate ratio analysis test was performed on Fab glycans excised from CHO-cetuximab. The released Fab glycans were precipitated with ice ethanol, the supernatant was centrifuged and spin-dried, and then labeled with *para*-aminobenzyl (2-AB). 2-AB labeling could make glycans have fluorescent detectable signals, and after reconstitution in 70% acetonitrile aqueous solution, was detected by UPLC coupled with a fluorescence detector (FLR). Good chromatographic peak separation was obtained using a hydrophilic interaction chromatography (HILIC) column. Thus, the test enabled stable glycan ratio analysis.

The molecular weight results for three independent Endo F2 digestion cycles for 5 min showed that the masses after digestion were similar; subsequently, glycan ratio analysis was performed based on HILIC. The results of three independent glycan ratio analysis experiments were also similar, indicating that the rapid enzyme digestion of Endo F2 followed by glycan ratio analysis after 5 min of digestion yielded good stability and reliability. Data obtained by measuring the samples produced using two different processes employed by our company showed that there were distinct differences in the glycan profiles of the two processes, especially in terms of the sialic acid glycoforms. These results prove that the method developed in this study can accurately analyze the ratio of glycans. Monitoring the antibody production process is important and meaningful for the evaluation of the process.

治疗性单克隆抗体(mAb)药物是以一类*γ*型免疫球蛋白为结构基础的大分子蛋白类药物,而西妥昔单抗(cetuximab,商品名爱必妥)是最早上市的被美国食品药品监督管理局(FDA)批准的抗表皮生长因子受体(EGFR)单克隆抗体,用于转移性结直肠癌和头颈鳞状细胞癌的治疗。默克公司生产的原研药采用的细胞系为小鼠骨髓瘤细胞(SP2/0),该细胞系在糖基化的表达过程中会形成*N*-羟乙基神经氨酸(NGNA)与*α*-半乳糖(*α*-Gal),据文献^[1,2]^报道,SP2/0表达的西妥昔单抗在临床上的副作用主要是出现严重的超敏反应,具体表现如呼吸困难、胸闷、皮肤瘙痒和视觉障碍等。而经中国仓鼠卵巢(CHO)细胞表达的西妥昔单抗,虽具有相同的氨基酸序列,但在糖基的组成和种类上存在很大差异,主要表现为形成*N*-乙基神经氨酸(NANA)的唾液酸化糖型修饰,且不再含有*α*-Gal,大大降低了潜在的免疫原性风险^[3,4,5]^。

截至目前,已上市的单克隆抗体药物绝大多数都是含有2个糖基化位点,且对称分布于两条重链上^[6,7,8]^。而已上市的西妥昔单抗是唯一一个具有4个糖基化位点的单抗药物,其中抗原结合片段(Fab)的2个糖基化位点位于高可变区(VH),可结晶片段(Fc)的2个糖基化位点位于恒定区2(CH2)。西妥昔单抗由SP2/0细胞表达,糖基化更加复杂,并伴随大量的唾液酸化,导致其具有显著的电荷异质性^[9,10]^。目前国内外许多研究者开始开发以CHO细胞表达的西妥昔单抗,相比于SP2/0细胞表达,在Fab和Fc段的糖基化修饰上会有比较大的差异,特别是Fab段,不再含有*a*-Gal和NGNA,取而代之的是大量的NANA。这种聚糖种类的变换以及聚糖含量不同的差异,对生物学活性及功能的影响,甚至在临床上的表现,还在进一步研究当中^[11,12,13,14]^。

本文以CHO细胞表达的西妥昔单抗为研究对象(CHO-cetuximab),从糖蛋白水平和聚糖水平对其糖基化修饰做了系列研究。主要利用*β*-*N*-乙酰氨基葡萄糖苷酶(Endo F2),结合质谱对质量数的归属分析,考察了抗体原液经Endo F2不同时间处理后的聚糖释放效果。结果显示,Endo F2对抗体Fab段的糖基化位点具有很高的酶切效率,能快速切除Fab段的聚糖,而Fc段的聚糖不受影响。对经Endo F2酶切下来的聚糖,进一步通过对氨基苯甲酰胺(2-AB)标记后使用亲水相互作用色谱柱(HILIC)分离,在超高效液相色谱-荧光检测器(UPLC-FLR)上实现了准确的聚糖比例分析。

## 1 实验部分

### 1.1 仪器、试剂与材料

CHO-西妥昔单抗为本公司经CHO稳定细胞系表达的西妥昔单克隆抗体原液。高分辨质谱仪Q-Exactive、蛋白质分析软件Biopharma Finder 3.2和液相系统Dionex U3000均购自Thermo Scientific公司(美国);超高效液相色谱Acquity UPLC H-Class plus系统、荧光检测器2475 FLR均购自Waters公司(美国);糖苷酶Endo F2购自Agilent公司(美国); *N*-肽糖苷酶F(PNGase F)购自NEB公司(英国);乙腈(ACN)、2-AB(纯度≥98%)购自Sigma-Aldrich公司(美国);甲酸胺购自Honey Well公司(美国);超纯水Milli-Q系统购自Millipore公司(美国)。

### 1.2 实验方法

1.2.1 释放抗体Fab和Fc聚糖

Endo F2酶切^[15]^:将抗体原液用超纯水稀释至1.0 mg/mL,然后取100 μL,加入Endo F 21.0 μL,快速混匀后于37 ℃下孵育16 h。取50 μL用于LC-MS分析,剩余50 μL加入150 μL预冷的冰乙醇,于-20 ℃条件下沉淀蛋白质,以13000 r/min离心5 min后取上清液用于聚糖的定性分析。

PNGase F酶切^[15,16]^:将抗体原液用8 mol/L盐酸胍变性稀释至1.0 mg/mL,然后取100 μL,加入PNGase F 2.0 μL,快速混匀后于37 ℃下孵育16 h。其余操作同Endo F2酶切。

1.2.2 Fab聚糖的快速释放以及荧光标记

将抗体原液用超纯水稀释至1.0 mg/mL,然后取100 μL,加1.0 μL Endo F2酶,快速混匀后于37 ℃下孵育5 min。取50 μL用于LC-MS分析,剩余50 μL加入150 μL预冷的冰乙醇,于-20 ℃放置40 min,沉淀蛋白质,以13000 r/min离心5min后取上清液,经真空浓缩干燥仪彻底干燥,加入2-AB标记液于65 ℃避光标记3 h(使聚糖具有荧光信号),用50 μL 70%乙腈水溶液复溶,然后进行UPLC-FLR聚糖比例的分析^[15]^。

1.2.3 LC-MS分析

采用Waters BioResolve RP mAb多苯色谱柱(100 mm×2.1 mm, 2.7 μm),流动相A和B分别为0.1%(v/v)FA水溶液和含0.1%(v/v)FA的乙腈溶液,流速0.4 mL/min。梯度洗脱程序为0~2 min, 10%B; 2~6 min, 10%B~90%B。

质谱采用正离子模式,喷雾电压设为3.8 kV,毛细管加热温度320 ℃,辅助气温度350 ℃,鞘气压力40 arb,辅助气压力10 arb,一级质谱扫描质荷比(*m/z*)范围1800~5000。采用BioPharma Finder 3.2软件对原始谱图进行去卷积化处理,*m/z*处理范围:2000~4000;输出质量数范围:130000~160000。

1.2.4 UPLC-FLR分析

采用Waters BEH Amide亲水色谱柱(150 mm×2.1 mm, 1.7 μm),流动相A为100 mmol/L甲酸铵溶液(pH 4.5),流动相B为乙腈,流速0.5 mL/min,柱温60 ℃。梯度洗脱程序为0~18 min, 2%A~22%A; 18~38 min, 22%A~44%A。荧光检测器激发波长330 nm,接收波长420 nm。

## 2 结果与分析

### 2.1 抗体Fab与Fc聚糖的释放与分析

糖苷酶PNGase F是抗体糖基化研究过程中一种常用的聚糖释放酶,能快速并完全释放聚糖并进行相应的定量研究,特异性地切断糖基化位点天冬酰胺(Asn)和与之相连*N*-乙酰葡糖胺之间的糖苷键,并将Asn转变为天冬氨酸(Asp)^[17,18]^。Endo F2也能实现聚糖的释放,能切断两个*N*-乙酰葡糖胺之间*β*-1,4键,释放截短的聚糖分子,同时另一个*N*-乙酰葡糖胺仍保留在糖基化位点的Asn上^[19,20]^。

CHO-西妥昔单抗蛋白分子为IgG1型,且共有4个糖基化位点,每条重链的Fab段和Fc段各有1个*N*-糖基化位点,可以通过糖苷酶PNGase F和Endo F2对其切糖,释放出相应的聚糖后进行糖相关分析(见图1)。

**图1:**
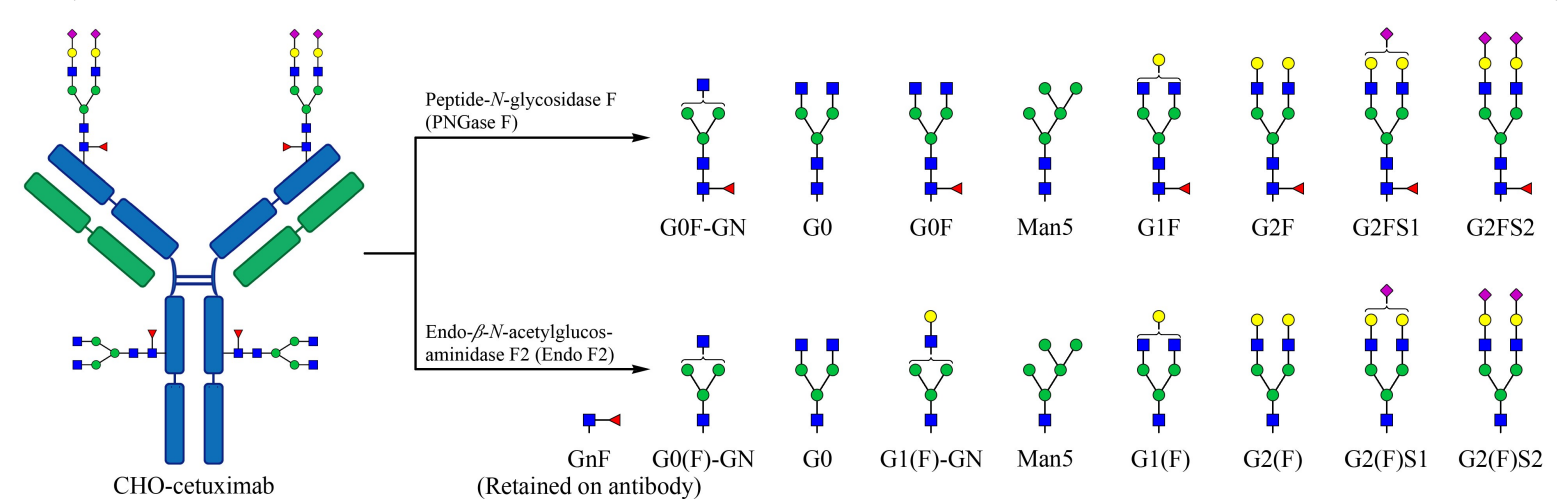
西妥昔单抗结构及释放的聚糖

将变性后的抗体经PNGase F酶切16 h后,使用LC-MS检测,经去卷积处理后(见图2a),测得酶切后抗体分子的质量数为145277.66(理论相对分子质量为145277.67 Da),说明抗体的聚糖已经被完全切除,同时糖基化位点的Asn转变为Asp。而未变性的抗体经Endo F2酶切16 h后(见图2b),测得酶切后抗体分子的质量数为146672.05(理论相对分子质量为146670.28 Da),说明抗体的聚糖也已经被完全切除,同时糖基化位点上均保留了GnF。以上结果表明,在相同的酶切时间16 h的条件下,Endo F2与PNGase F的变性酶切均能实现聚糖的完全释放。将释放出来的聚糖,在HILIC柱上分离并与质谱进行联用分析,可以实现对抗体全部聚糖的鉴定,可以看到CHO-西妥昔单抗上有较多的唾液酸化糖型G2FS1和G2FS2,此外两种酶切方式产生的糖谱非常相似(见图3),说明对聚糖的定性和定量具有一致性的结果。

**图2:**
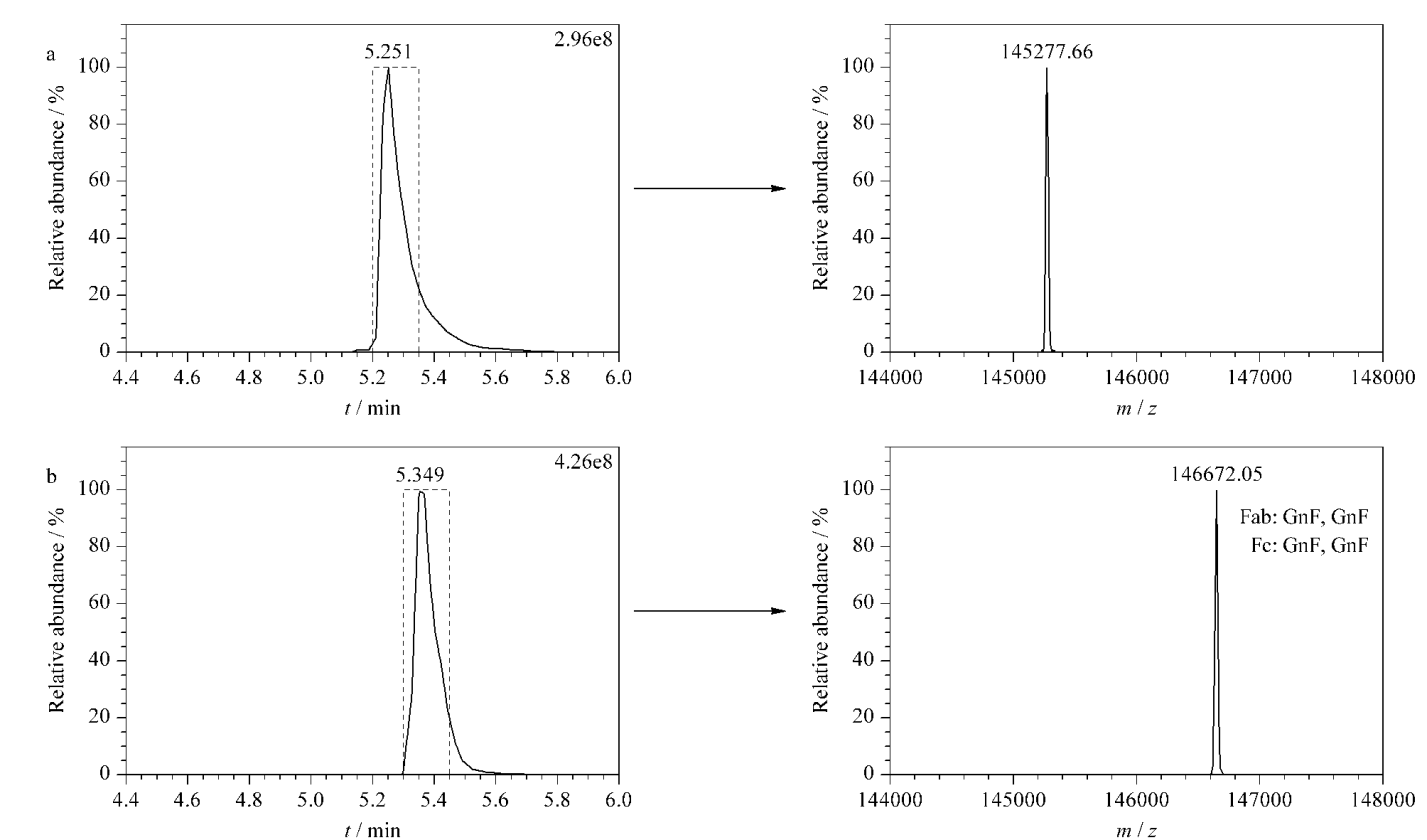
糖苷酶处理16 h后的TIC色谱图和去卷积谱图

**图3:**
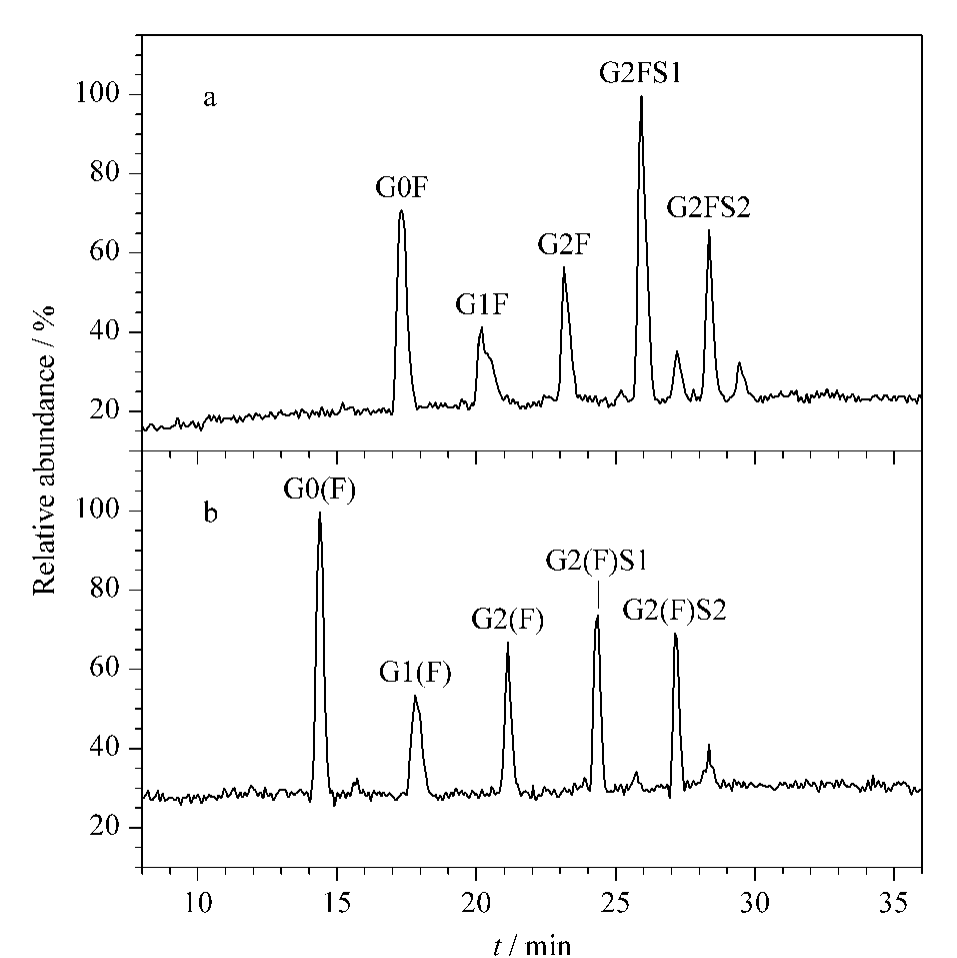
聚糖的TIC色谱图

在以往对西妥昔单抗的糖基化研究中发现,PNGase F在非变性条件下能切除Fc段Asn 299位置的糖基,但Fab段的Asn 88位置的糖基依然存在,而Endo F2能在非变性条件下切除Fab段的糖基^[18]^。这种选择性的切除方式推测是与抗体的空间结构有关,糖苷酶在进攻糖基化位点时需要突破空间位阻才能进行切糖。在本实验中我们发现,使用Endo F2在非变性条件下处理CHO-西妥昔单抗,只要酶切时间足够长,Endo F2既能完全切除Fab段的糖基,也能完全切除Fc段的糖基。我们推测抗体的空间位阻效应降低了Endo F2对Fc段糖的酶切效率,但无法阻止其对Fc段糖的酶切,因此我们后续考察了Endo F2的不同酶切时间。

### 2.2 Endo F2酶切时间的考察

经Endo F2酶切5 min(见图4a),可以看到只有Ⅰ类质量数,通过对比理论分子量对照表(见附表S1,详见http://www.chrom-China.com),确定为抗体的Fab段2个位点均被切除:Fab段聚糖被切除后均保留了岩藻糖化的*N*-乙酰葡萄糖胺(GlcNAc)残基(GnF), Fc段上2个位点保留了G0F、G1F、G2F之间的聚糖组合。说明Endo F2对Fab段的酶切效率非常高,5 min就已经将抗体Fab段2个位点上的聚糖完全切除,而Fc段的聚糖未受影响。酶切30 min后可以看到除了有Ⅰ类的质量数,还有Ⅱ类的质量数(见图4b), Ⅱ类质量数确定为抗体的Fab段2个位点均被切除和Fc段1个位点被切除:Fab段的2个位点和Fc段的1个位点被切除后均保留了GnF,而Fc段还有1个位点保留了G1-GN、G0F、G1F、G2F。说明酶切时间至30 min时,有一部分抗体的Fc段中1个位点上的聚糖开始被切除了。进一步延长酶切时间至300 min后(见图4c),可以看到有Ⅰ、Ⅱ、Ⅲ类质量数,Ⅲ类质量数确定为抗体的Fab段2个位点和Fc段2个位点均被切除:4个位点上均保留了GnF,相对应的质量数为146670.08。得出Endo F2对Fc段的酶切效率相对较低,酶切300 min后仍然有较多Fc聚糖未完全切除的抗体,若要完全切除Fc段的聚糖则需要更长的时间,如本实验中的16 h。

**图 4:**
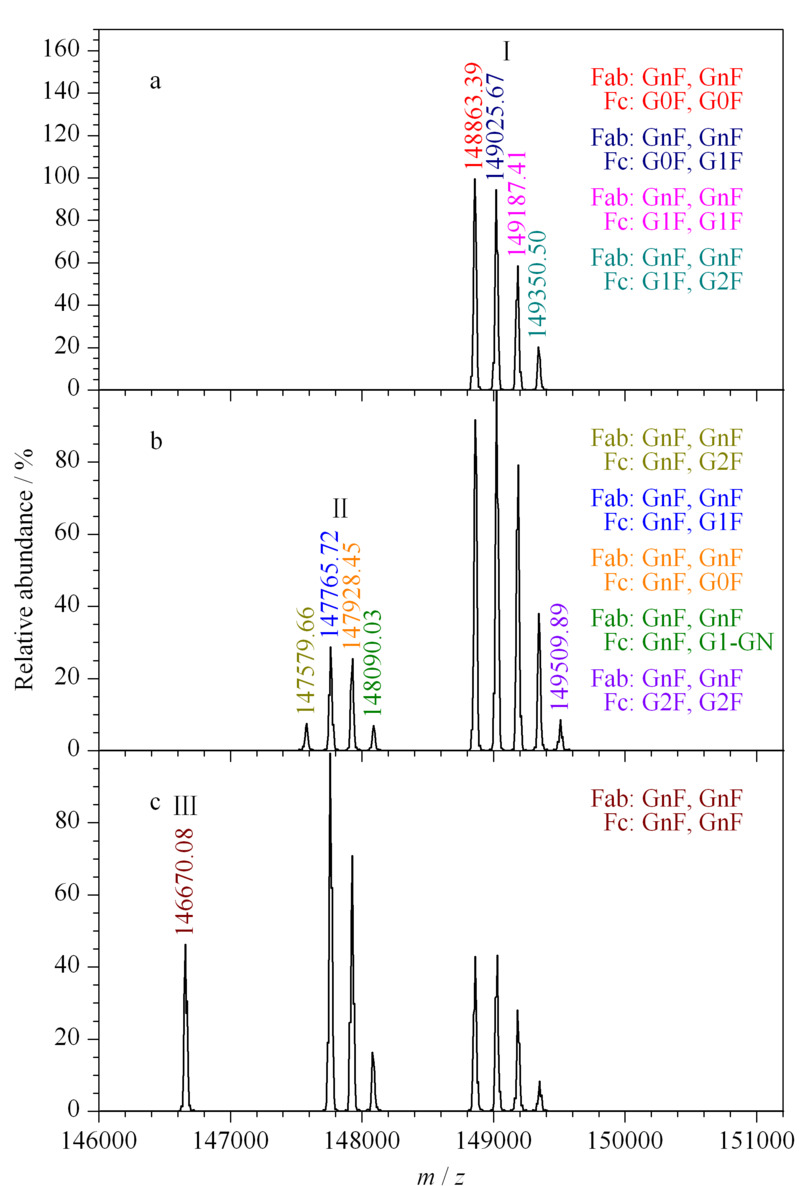
Endo F2不同酶切时间的质谱图

抗体药物的生产工艺非常复杂,而且批次间的质量属性可能会有一些波动,尤其是糖型方面,对西妥昔单抗这种具有复杂糖基化位点的药物,分析各个位点上的糖基化显得尤为重要。从本实验的结果来看,使用Endo F2酶切5 min,抗体的Fab段2个位点的聚糖完全被切除,说明该条件下可以专一性地切除Fab段位点上的聚糖,本实验也因此未做更短酶切时间的考察。抗体经Endo F2酶切后,后续可以通过UPLC-FLR准确分析Fab段位点上的聚糖比例,对抗体生产工艺的评估具有非常重要的意义。

### 2.3 UPLC-FLR对Fab段聚糖的比例分析

从糖蛋白上释放的聚糖经2-AB衍生化后,使用HILIC色谱柱在UPLC-FLR上可以实现聚糖的比例分析。CHO-西妥昔单抗抗体经Endo F2酶切5 min,释放的聚糖经2-AB标记后,在HILIC色谱柱上具有良好的分离效果(见图5),结果展示了Fab段的糖基化修饰结果,图5a和图5b的样品产自两个不同的生产工艺,分别是工艺Ⅰ和工艺Ⅱ,其中工艺Ⅱ是在工艺Ⅰ基础上,在CHO细胞培养过程中额外加入了适量的糖型调节剂,从而调节了抗体的糖型。从结果可以看出,经不同生产工艺表达的CHO-西妥昔单抗在Fab段的糖谱上具有非常明显的差异。其中还可以看出含唾液酸化的聚糖比例也相差较大,工艺Ⅰ的G2(F)S1和G2(F)S2的总比例较低,约25.0%,而工艺Ⅱ的总比例较高,约41.4%。此外,为了考察本方法的稳定可靠性,我们独立做了3次试验,工艺Ⅱ样品的3次Endo F2酶切后的相对分子质量基本一致(见附图S1), RSD值几乎为0, 3次试验的聚糖在HILIC色谱柱上的比例分析谱图也基本一致(见附图S2),峰面积的RSD值均小于5.0%,表明本方法具有较好的稳定性。

**图5:**
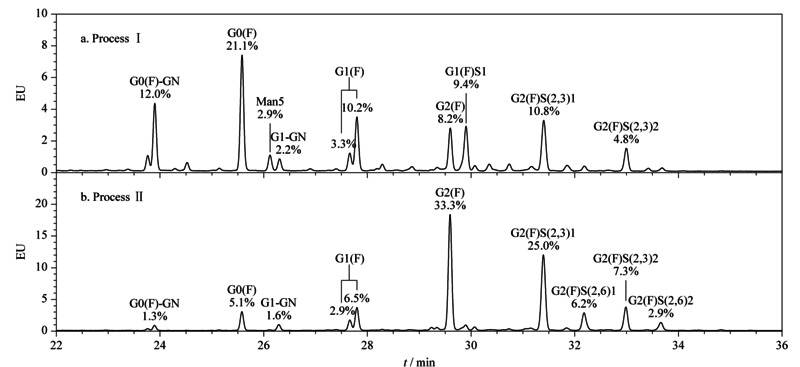
来自不同生产工艺的CHO-西妥昔单抗抗体Fab段的糖谱

抗体的Fab区是识别抗原的关键区域,西妥昔单抗Fab上的糖基化位点处在抗体重链的可变区,该位点上的糖基化可能参与免疫调节,影响抗原抗体的亲和力,因此对该位点的糖基化修饰分析具有非常重要的意义。通过Endo F2非变性酶切5 min,结合2-AB标记的方法可以实现对Fab段聚糖的糖型比例分析,对指导抗体生产工艺的优化起到非常大的帮助。

## 3 结论

本文针对CHO细胞表达的西妥昔单抗,开发了Endo F2快速酶切结合UPLC-FLR检测的糖型分析方法,不仅对Fab段糖基化修饰的分析具有很好的专一性,而且还具有较好的稳定可靠性,大大提高了检测效率。这对研发者研究具体的糖型构成,抗体抗原的亲和力等提供了数据支撑,对经不同细胞表达的,以及经不同的细胞培养工艺生产的西妥昔单抗,研究Fab段糖型的变化和电荷异质性提供了一种非常方便、快捷、准确的分析手段,并能够有效指导生产工艺的优化以及后续工艺可比性的研究。
